# Impact of Neurodynamic Sequencing on the Mechanical Behaviour of the Median Nerve and Brachial Plexus: An Ultrasound Shear Wave Elastography Study

**DOI:** 10.3390/diagnostics14242881

**Published:** 2024-12-21

**Authors:** Gianluca Ciuffreda, Elena Estébanez-de-Miguel, Isabel Albarova-Corral, Miguel Malo-Urriés, Michael Shacklock, Alberto Montaner-Cuello, Elena Bueno-Gracia

**Affiliations:** 1 Department of Human Anatomy and Histology, Faculty of Health Sciences, University of Zaragoza, Calle Domingo Miral S/N, 50009 Zaragoza, Spain; 2 PhysiUZerapy: Health Sciences Research Group, University of Zaragoza, Calle Domingo Miral S/N, 50009 Zaragoza, Spain; ialbarova@unizar.es (I.A.-C.); malom@unizar.es (M.M.-U.); albertomontaner@unizar.es (A.M.-C.); ebueno@unizar.es (E.B.-G.); 3 Department of Physiatry and Nursing, Faculty of Health Sciences, University of Zaragoza, Calle Domingo Miral S/N, 50009 Zaragoza, Spain; 4 Neurodynamic Solutions, Adelaide 5034, Australia; shacklock@yahoo.com

**Keywords:** neurodynamics, biomechanics, median nerve, brachial plexus, nerve roots, elastography

## Abstract

Background: When performing the Upper Limb Neurodynamic Test 1 (ULNT1), the order of joint movement can be varied to place more stress onto certain nerve segments. However, the mechanisms underlying this phenomenon are still unclear. This study aimed to analyze the differences in the stiffness of the median nerve (MN) and the brachial plexus (BP) using ultrasound shear wave elastography during three sequences of the ULNT1: standard (ULNT1-STD), distal-to-proximal (ULNT1-DIST), and proximal-to-distal (ULNT1-PROX). Methods: Shear wave velocity (SWV) was measured at the initial and final position of each sequence at the MN (wrist) and at the C5 and C6 nerve roots (interscalene level) in 31 healthy subjects. Results: A significant interaction was found between ULNT1 sequence and location (*p* < 0.001). The ULNT1-STD and ULNT1-DIST induced a greater stiffness increase in the MN (5.67 ± 0.91 m/s, +113.94%; 5.65 ± 0.98 m/s, +115.95%) compared to C5 and C6 (*p* < 0.001). The ULNT1-PROX resulted in a significantly smaller increase in stiffness at the MN (4.13 ± 0.86 m/s, +54.17%, *p* < 0.001), but a greater increase at C5 (4.88 ± 1.23 m/s, +53.39%, *p* < 0.001) and at C6 (4.87 ± 0.81 m/s, +31.55%). The differences for the ULNT1-PROX at C6 were only significant compared to the ULNT1-STD (*p* < 0.001), but not the ULNT1-DIST (*p* = 0.066). Conclusions: BP and MN stiffness vary depending on the joint movement sequence during neurodynamic testing. However, the influence of the surrounding tissues may have affected SWV measurements; consequently, these results should be interpreted with caution.

## 1. Introduction

Neurodynamic tests (NDTs) are tests widely used to assess the dynamics (e.g., movement and sensitivity) of neural structures and diagnose peripheral neuropathies [1,2,3,4,5,6]. NDTs consist of performing a series of movements that elongate the nerve bed, producing sliding and tension of the nerve along the neural tract [7,8,9,10,11,12,13].

It has been previously observed that varying the movements used during NDTs can produce changes in nerve behavior [14,15,16,17,18,19,20,21,22]. This phenomenon has been called “neurodynamic sequencing” [18,23]. Specifically, it has been suggested that the joint that moves first places more stress on the region of the nerve closest to that joint. Conversely, the area of the nerve furthest from the joint where the NDT begins receives less stress during the test [18,19,20,23]. If this were true, the sequence of movements could be adapted to the patient’s clinical situation for improvements in diagnosis and treatment.

The Upper Limb Neurodynamic Test 1 (ULNT1) is used to evaluate the median nerve and brachial plexus [4,6,14,24,25,26]. The ULNT1 moves the joints of the upper extremity and neck to apply tension to the median nerve and brachial plexus, so the evoked response can be analyzed for diagnosis [6,24,27,28,29]. Clinical studies have shown that neurodynamic sequencing modifies both the area of symptoms reported by patients during NDT and the final position of the test (differences in the final ROM of each joint) in asymptomatic subjects [18,20] and carpal tunnel syndrome patients [14]. Furthermore, neurodynamic sequencing has also been found to improve ULNT1 diagnostic accuracy for carpal tunnel syndrome [30]. However, the mechanisms underlying this phenomenon are still unclear.

Studies performed on cadaveric specimens have shown contradictory results [17,19]. Tsai (1995) [19] showed that strain in the ulnar nerve at the elbow was 18% greater than other sequences of movement when elbow flexion occurred first. However, Nee et al. [17] showed that the strain and excursion of the median nerve at the wrist was similar between sequences except when the nerve started movement earlier when the wrist was moved first. This suggested that strain may not be as relevant as originally thought, and instead, duration at the end position might be more important [17].

Shear wave elastography (SWE) has emerged as a useful tool for the assessment of various peripheral neuropathies [31,32,33,34] and has been shown to be suitable to measure in vivo nerve stiffness with joint movement [22,35,36,37]. Shear wave elastography reflects the stiffness of the nerve by measuring the speed of a shear wave produced in the tissue after the emission of an acoustic pulse [33,38]. More recently, SWE has been used to measure changes in median nerve (MN) stiffness during variations in the ULNT1 movement sequence [22].

Ciuffreda et al. [22] showed that performing the ULTN1 with three different movement sequences produced changes in MN stiffness. Specifically, the standard sequence (as described by Shacklock [23]) and the distal-to-proximal sequence, which began with wrist extension, produced a greater increase in MN stiffness at the wrist compared to the proximal-to-distal sequence, which started with contralateral cervical lateral flexion. A notable aspect is that changes in MN stiffness were observed at the wrist but not at the elbow, possibly due to the anatomical location of the elbow in the mid-portion of the upper extremity rather than the proximal region [22]. The proximal-to-distal sequence may have produced different stiffness values in more proximal segments of the neural tract, such as the brachial plexus, but no measurements were performed in this region [22].

Therefore, the objective of the present study is to analyze and quantify differences in the stiffness of the MN at the wrist and the brachial plexus (BP) at the neck by measuring the shear wave velocity (SWV) with ultrasound elastography during three sequences of the ULNT1, i.e., standard (ULNT1-STD), distal-to-proximal (ULNT1-DIST), and proximal-to-distal (ULNT1-PROX) sequences.

## 2. Materials and Methods

A cross-sectional study was conducted in a physiotherapy clinic in Zaragoza (Spain) following the STROBE guidelines [39]. The study was approved by the local Institutional Ethical Committee (CEICA, reference: C.I. PI23/340), and all procedures were conducted in accordance with the Declaration of Helsinki (last modified in 2013) [40]. Prior to the collection of data, informed written consent was obtained from all participants.

### 2.1. Sample

Thirty-one asymptomatic subjects were recruited through social media advertisements and informational posters from March to July 2024.

Eligibility criteria included being aged 18 to 65, having full passive range of motion in all joints involved in ULNT1 sequences, and showing symptoms within 120° to 170° of elbow extension in the ULNT1-STD [10,23]. Participants were excluded if they reported pain, altered sensitivity, paresthesia or dysesthesias, weakness in the spinal column or upper extremity in the past year, any recent injuries to the thoracic or cervical spine and/or upper extremity, autoimmune diseases, diabetes, thyroid disorders, or central nervous system disorders. Shear wave elastography measurements were performed only when a ULNT1 sequence showed positive structural differentiation (i.e., a change in symptoms at the end of the test related to a structural differentiation maneuver, suggesting neural tissue involvment in the response) [4,6,10,11,23,27,41,42,43,44,45,46]. Participants provided informed, written consent before the study. After obtaining consent, baseline demographic information, including sex, age, height, weight, and body mass index (BMI), was recorded.

A power analysis was conducted using G*Power 3.1 (Faul, Erdfelder, Lang, and Buchner, 2007) [47] with the data obtained from the initial thirty-one participants. The power analysis was calculated for a two-way repeated measures ANOVA [sequence/position × location] using the “SWV” and the “ % of increase in SWV from the starting position” as dependent variables. The partial η^2^ value considered for the calculation was 0.543, which was the lowest effect size obtained among both variables. With α set at 0.05 and a sample size of 31, the statistical power obtained was 1. Consequently, it was determined that no further participants were required.

### 2.2. Ultrasound Procedures

Imaging and SWE were carried out using a LOGIQ P9 ultrasound system (GE Healthcare, Chicago, IL, USA) with a high-frequency linear transducer L3-12 (12 MHz).

Briefly, SWE is an ultrasound-based technique aimed to assess the stiffness of a determined tissue by quantifying the propagation velocity of the shear wave generated from an acoustic pulse [33,38]. Assuming an elastic, homogeneous, and isotropic medium, the speed of the shear wave is directly linked to the shear modulus (µ) and Young’s modulus (E) through the following relationship: E = 3µ = 3ρc^2^, where ρ is the density (assuming ρ = 1000 kg/m^3^), and c is the shear wave speed [48].

During SWE, the system’s predefined spatial and temporal filters for musculoskeletal applications “Nerve” (spatial filter 5, temporal filter 4, spatial resolution of 1 mm) were selected. Left MN and BP were imaged immediately proximal to the wrist crease at the quadratus pronator level and between the anterior and middle scalene muscles, respectively. For an optimal visualization of the ultrasound image, the required amount of conductive ultrasound gel was used. To minimize compression on the underlying tissues during the scan, special care was taken to apply minimal pressure with the probe on the subject’s skin. The B-mode was used to identify MN and BP, following the procedure described below.

#### 2.2.1. Median Nerve Imaging

The median nerve was visualized longitudinally in the center of the ultrasound image immediately proximal to the wrist crease. To locate the median nerve, a transverse scan was performed along its length to identify its honeycomb structure. Once the nerve was identified in the transverse plane, the probe was rotated 90° to obtain the longitudinal image [22,36].

#### 2.2.2. Brachial Plexus Imaging

The C5 and C6 nerve roots of the brachial plexus were first identified in the transverse plane at the interscalene level. The probe was then positioned immediately lateral to the thyroid lobe. The anterior and middle scalene muscles were identified, and the nerve roots of the brachial plexus were observed between them as hypoechoic oval structures. The C5 and C6 nerve roots were located between the anterior and posterior tubercles of the transverse process of their respective vertebrae. Once each root was visualized in the transverse plane, the longitudinal plane was obtained by rotating the probe 90° [49,50].

#### 2.2.3. Shear Wave Elastography and Ultrasound Measurements

**Figure 1:**
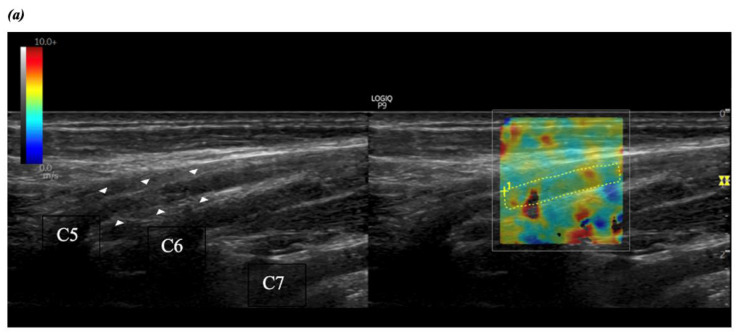

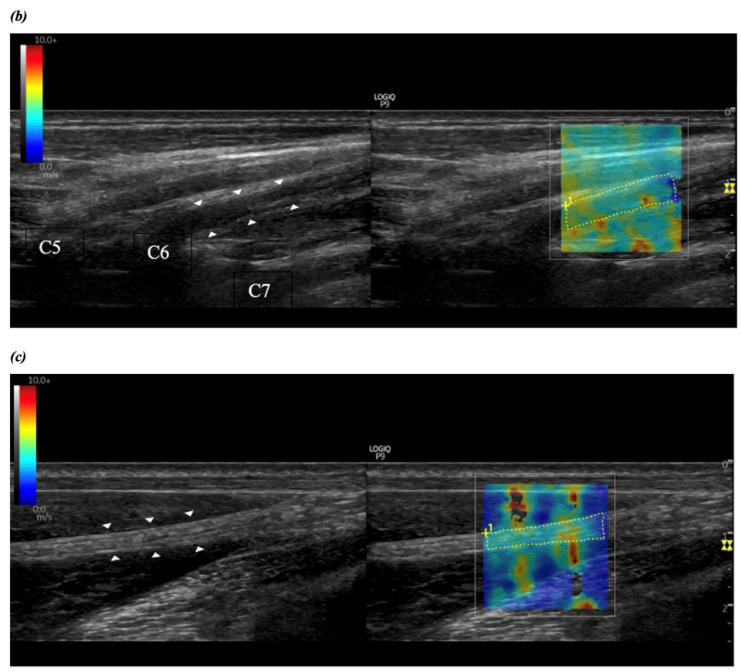
Shear wave elastography of the brachial plexus at the level of C5 (**a**) and C6 (**b**) nerve roots and median nerve at pronator quadratus (**c**). White arrowheads: C5 (**a**), C6 (**b**) nerves roots, or median nerve (**c**). The discontinuous yellow lines indicate the region of interest (ROI), and the shear wave velocity (SWV) is expressed in meters per second (m/s). B-mode gray scale: from black (anechoic) to white (hyperechoic). SWV color scale: from dark blue (0 m/s) to red (10 m/s).

Shear wave elastography was conducted in dual mode, in which the elastogram was displayed alongside the gray-scale image. A 2.0 × 2.0 cm elastography box was selected to include MN and C5–C6 roots with their surrounding tissues, avoiding as many bony regions as possible. The elastogram displayed tissue shear wave velocity (SWV) on a color scale from 0 (dark blue) to 10 m/s (red). SWV values were automatically calculated for each image from a manually outlined region of interest (ROI), covering the entire nerve area within the elastography box (Figure 1). The average SWV from the three measurements was used for analysis.

The thickness and angle across the image were measured in B-mode for MN, C5, and C6 nerve roots. The thickness was measured by tracing a straight line within the epineurium boundaries in the mid-portion of the neural structure included in the elastography box. The angle was calculated by measuring the degrees between the longitudinal axis of the nerve segment in the elastography box and a horizontal line across the ultrasound image.

All ultrasound data were collected by the same examiner (G.C.). Room temperature was maintained at 21 °C.

### 2.3. ULTN1 Sequences

Measurements were performed in both the starting (P0) and ending (P1) positions of the ULNT1-STD, ULNT1-PROX, and ULNT1-DIST.

At P0, participants lay on their backs with their neck and shoulders in a neutral position, 30° of glenohumeral abduction in neutral rotation, 90° of elbow flexion, and forearm, wrist, and fingers in a neutral position. An examiner (I.A.-C.) then performed the ULNT1 sequences in the upper left extremity in random order with at least 3 min rest between each test [5]. For all ULNT1 sequences P1 were determined by symptoms’ onset.

The movements for each sequence were as follows:

(a) ULNT1-STD [23]:
  (1) Shoulder abduction from 90° to 110°;
  (2) Shoulder external rotation to the frontal plane;
  (3) Forearm supination;
  (4) Wrist and finger extension;
  (5) Elbow extension.
(b) ULNT1-PROX:
  (1) Cervical contralateral lateral flexion;
  (2) Shoulder abduction from 90° to 110°;
  (3) Shoulder external rotation to the frontal plane;
  (4) Elbow extension;
  (5) Forearm supination;
  (6) Wrist and finger extension.
(c) ULNT1-DIST:
  (1) Wrist and finger extension;
  (2) Forearm supination;
  (3) Elbow extension;
  (4) Shoulder external rotation to the frontal plane;
  (5) Shoulder abduction.

To confirm the neural origin of symptoms, structural differentiation was performed in all three ULNT1 sequences [4,6,10,11,23,27,41,42,43,44,45,46]; for distal symptoms, neck or shoulder girdle was employed, while for proximal symptoms, wrist movements were used. If the maneuver modified symptoms, structural differentiation was considered positive (i.e., neural tissue is implicated), whereas a lack of symptom change suggested a musculoskeletal origin [4,6,10,11,23,27,41,42,43,44,45,46].

At P1 of all sequences, the range of motion (ROM) of the last joint moved was measured by a third examiner (A.M.-C.) using a standard 360° two-arm goniometer. For elbow extension, the axis of the goniometer was located in alignment with the medial epicondyle of the humerus, with the stationary arm pointing towards the acromion and the moving arm aligned with the ulnar styloid [10,51]. For shoulder abduction, the goniometer was positioned at the acromion, with the stationary arm parallel to the sternum and the moving arm aligned with the medial epicondyle of the humerus [52,53].

### 2.4. Reliability of SWE

The intra-rater reliability was studied in 20 upper extremities from ten participants (age: 30.10 ± 9.15 years; 6 females and 4 males). Two assessments were performed by the same examiner (G.C.) at the MN and BP at P0 and P1 of each ULNT1 sequences. Reliability was calculated with a two-way mixed effect intraclass correlation coefficient (ICC) “absolute agreement” [54]. The standard error of measurement (SEM) and the minimal detectable change (MDC) were calculated using the following formulae: SEM = standard deviation × $\sqrt{\left(1-ICC\right)}$; MDC = SEM × 1.96 × $\sqrt{2\;}$. The mean SWV obtained from three elastographies was used for the analyses.

### 2.5. Statistical Analysis

Statistical analysis was carried out using the SPSS 29.0 software (SPSS Inc., Chicago, IL, USA). The Shapiro–Wilk test was used to check for the normal distribution of data. The mean ± standard deviation (SD) was used to express quantitative data, unless otherwise specified. For all SWV analysis, the average value obtained from three elastographies was used. A two-way repeated measures ANOVA [position × location] was conducted using the SWV, its percentage of increase from P0, neural thickness, and angle as dependent variables. The Mauchly test was used to assess sphericity in all ANOVA analyses; if violated, the degrees of freedom were adjusted using Greenhouse–Geisser correction. Bonferroni corrections were applied for post hoc comparisons. Correlations between SWV, thickness, and angle across the image were investigated using Spearman’s correlation coefficient (r_s_). α level was set at 0.05.

## 3. Results

Thirty-three volunteers were initially selected for eligibility. Two subjects were excluded as they presented less than 120° of ROM during the ULNT1-STD. Thus, the final sample consisted of 31 healthy participants (age: 36.06 ± 11.98 years; gender: 20 females and 11 males; dominant side: twenty-eight right and three left; height: 171 ± 10 cm; weight: 70.16 ± 13.74 kg; body mass index: 23.74 ± 3.25). All ULNT1 sequences showed a positive structural differentiation; therefore, SWV was assessed in all tests.

SWE reliability for the assessment of MN and BP is summarized in Table 1.

### 3.1. Median Nerve and Brachial Plexus Shear Wave Velocity

**Figure 2:**
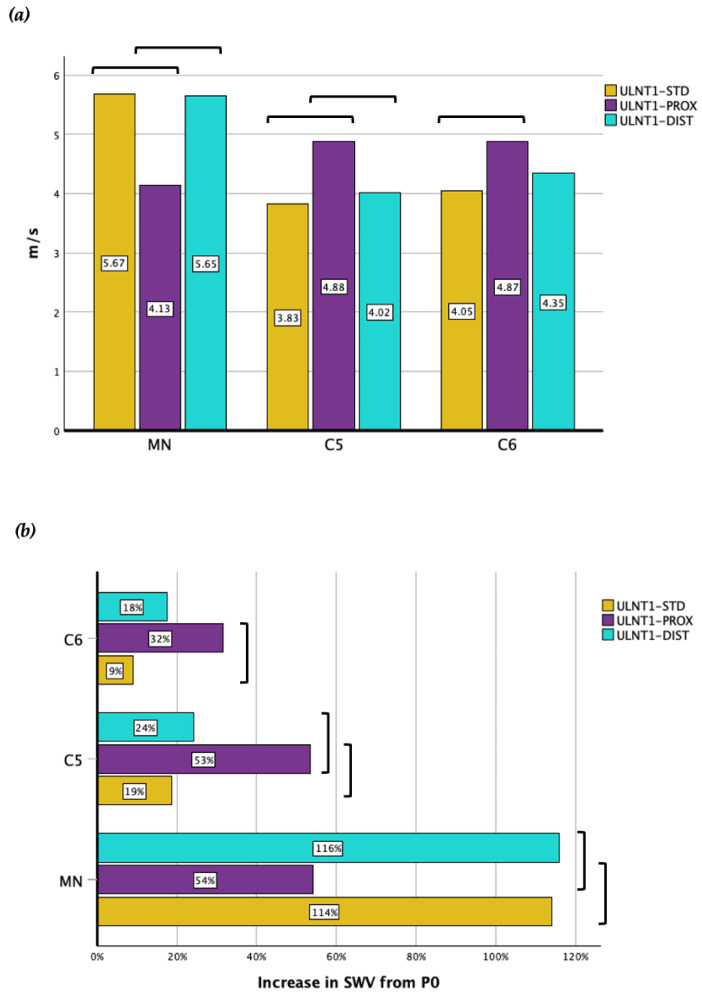
Median nerve and brachial plexus. (**a**) SWV at the end point of each ULNT1 sequence, and (**b**) SWV increases from P0. Bars indicate statistically significant difference when comparing two sequences at the same location.

MN and BP SWV data are summarized in Table 2 and Figure 2.

Two-way repeated measure ANOVA revealed a significant interaction between ULNT1 sequence and location for both mean SWV and its percentage of change from P0 (*p* < 0.001).

At P0, mean SWV was significantly lower in the MN (2.76 ± 0.61 m/s) compared to C5 (3.28 ± 0.69 m/s, *p* = 0.007) and C6 (3.77 ± 0.55 m/s, *p* < 0.001). In the same position, the difference between C5 and C6 was also statistically significant (*p* < 0.001).

All ULNT1 sequences led to a significative increase in SWV with respect to P0 in the MN and BP (ULNT1-STD at C5, *p* = 0.002; ULNT1-DIST at C6, *p* = 0.031; rest of the sequences and locations, *p* < 0.001), except for the ULNT1-STD in C6 (*p* = 0.468).

Similar SWV values were found in the MN for the ULNT1-STD and ULNT1-DIST (5.67 ± 0.91 m/s, +113.94% ± 50.27 increase; 5.65 ± 0.98 m/s, +115.95% ± 61.82 increase; *p* = 1.000) in C5 (3.83 ± 0.83 m/s, +18.76% ± 25.57 increase; 4.02 ± 0.86 m/s, +24.14% ± 22.83 increase; *p* = 1.000) and C6 (4.05 ± 0.82 m/s, +8.98% ± 23.61 increase; 4.35 ± 0.98 m/s, +17.65% ± 29.92 increase; *p* = 0.963). However, the ULNT1-STD induced a greater change in SWV in MN, compared to C5 and C6 nerve roots (*p* < 0.001 both), with values at P1 significantly greater with respect to C5 and C6 (*p* < 0.001 both). Analogously, the ULNT1-DIST sequence produced a larger increase in SWV in MN compared to C5 (*p* < 0.001) and C6 (*p* < 0.001), with higher SWV values at P1 (*p* < 0.001).

The ULNT1-PROX sequence led to a significantly smaller increase in SWV at the MN (4.13 ± 0.86 m/s, +54.17% ± 37.50 increase) compared to both the ULNT1-STD and ULNT1-DIST sequences (*p* < 0.001 each). In contrast, the ULNT1-PROX demonstrated an opposite effect at the BP. Specifically, at C5, a greater increase occurred (4.88 ± 1.23 m/s, +53.39% ± 46.14 increase) compared to both ULNT1-STD (*p* < 0.001) and ULNT1-DIST (*p* = 0.003). At C6, ULNT1-PROX also produced the greatest increase in SWV (4.87 ± 0.81 m/s, +31.55% ± 26.80 increase); however, this difference was only statistically significant when compared to ULNT1-STD (*p* < 0.001) but not ULNT1-DIST (*p* = 0.066).

Significant differences in SWV at P1 were also observed for the ULNT1-PROX between MN and C5 (*p* = 0.021) and between MN and C6 (*p* = 0.002).

At the BP level, all ULNT1 sequences produced similar SWV values at P1 in both nerve roots (*p* > 0.05 for each comparison); however, the ULNT1-PROX led to a higher increase from P0 in C5 (*p* = 0.039).

### 3.2. Final Position and Range of Motion

Table 3 shows the ULNT1 final positions and the ROM of the last joint moved. All participants in the ULNT1-STD and ULNT1-PROX reported responses during elbow extension, with an average ROM of 134.45° ± 13.75 and 136.00° ± 19.18, respectively. In the ULNT1-DIST, 30 subjects reported responses during glenohumeral abduction, with a mean ROM of 50.83° ± 14.38, and one subject at 155° of elbow extension.

### 3.3. Thickness and Angle Across the Image

Nerve thickness and angle values are listed in Table 2.

No significant interaction between positions and locations was found for nerve thickness (*p* = 0.238) or angle across the image (*p* = 0.122).

In all neural structures the ULNT1 sequences did not significantly alter nerve thickness or angle, except for a 0.292 mm difference found in nerve thickness at MN between P0 and ULNT1-STD (*p* = 0.006) and a 1.91° difference in nerve angle at C5 comparing ULNT1-STD with ULNT1-PROX (*p* = 0.030).

At the end of the ULNT1-DIST and ULNT1-PROX, C6 was thicker than C5 and MN (*p* < 0.05 each). No significant differences in thickness were found among neural structures in other positions. C5 and C6 showed a similar angle at P0 (*p* = 0.330), while in all other positions, C6 showed a significantly greater angle compared to both C5 and MN (*p* < 0.05 for each comparison), and C5 compared to MN (*p* < 0.05 for each comparison).

No correlation was found between SWV and nerve thickness (r_s_ = 0.092; *p* = 0.077). The angle across the image was weakly negatively correlated with SWV (r_s_ = −0.148; *p* = 0.004).

## 4. Discussion

The present study aimed to investigate the effect of the three sequences of the ULNT1 on BP and MN stiffness measured with ultrasound SWE. The findings suggest that the mechanical behavior of the BP and MN vary with the order of joint movement during neurodynamic testing.

The ULNT1-PROX resulted in the greatest SWV in the BP and the smallest in the MN. In contrast, the SWV values observed applying the ULNT1-STD and ULNT1-DIST sequences were similar, with both producing significantly higher SWV distally at the MN. Despite the ULNT1-PROX producing the greatest SWV values at C6, differences were significant only when compared to the ULNT1-STD. Previous studies have reported that wrist extension significantly increases SWV in the distal portion of the MN, regardless of whether the elbow is in a flexed or extended position [35,37]. Taking this into account, the non-significant differences in MN SWV between the ULNT1-STD and ULNT1-DIST sequences may be due to wrist extension, which was completed in both cases. In contrast, wrist extension could not be fully achieved in the ULNT1-PROX sequence due to the earlier onset of a neural response, which likely resulted in lower SWV values for the MN. Our results are consistent with previous research reporting similar SWV behavior for the MN at the wrist using the same ULNT1 sequence variations [22]. The ULNT1-PROX sequence involved maximal cervical contralateral lateral flexion, whereas in the ULNT1-STD and ULNT1-DIST sequences, the cervical spine remained neutral. This difference in cervical positioning may have mediated the SWV variations observed at the BP among these sequences. Overall, these results suggest that neural SWV may increase when a localized component of tissue tension is introduced in the ULNT1 sequence. The fact that ULNT1-PROX and ULNT1-DIST produced significantly different SWV increases at C5 but not at C6 could mean that distinct portions of the BP behave differently with variations in neck and limb movements.

Considering that movements aimed to increase neural tension are associated with greater nerve SWV [35,36,37], the differences in SWV observed between BP and MN with the three ULNT1 sequences suggest that mechanical loads do not build up uniformly along the neural tract and could vary depending on joint included as well as the movement order.

All ULNT1 sequences resulted in an increase in nerve SWV from the starting position across all tested locations. However, the increase at the C6 nerve root during the ULNT1-STD sequence was not significant. Considering that C6 has previously been shown to be subjected to more strain than C5 during different NDTs [55,56], a consequent larger increase in SWV at C6 would have been expected with respect to C5. One possible explanation for this discrepancy is the higher baseline SWV observed at C6 compared to C5 and the MN, which may suggest that the starting position did not equally unload these structures. In line with this, previous studies reported that shoulder abduction with a 90° flexed elbow does not significantly affect MN SWV distally [37], and that C5 strain seems to be less influenced by upper limb movements than other roots, such as C6–C8 [55,56]. Consequently, the slight amount of shoulder abduction at P0 could have applied more pre-tension of C6 compared to C5 and MN, which could partially explain the smaller SWV change observed from the baseline at the C6 level with the ULNT1-STD.

It must also be considered that C6 showed greater angles than C5 at the end of each sequence but similar values at P0; thus, the influence of tissue anisotropy must be considered when comparing SWV among the mentioned neural structures. This may account for the non-significant difference between C5 and C6 during the ULNT1-STD and ULNT1-DIST sequences, despite evidence that C6 nerve roots experience more strain during neurodynamic testing [55]. Nevertheless, further research is needed to fully understand nerve roots responses to various combination and angles of limb movements.

Previous studies in cadavers have shown that the order of joint movements during NDTs does not significantly affect the strain or excursion experienced by peripheral nerves [15,17]. However, in these studies, the same final position was reached at the end of all tests, while in our study, all ULNT1 sequences led to different joint angles. When a NDT is performed in vivo, the neural load is progressively increased as joint movements are added, and the test is typically concluded when symptoms appear [4,23,42,57]. Early movements in the sequence tend to have larger ranges of motion, while later movements are often restricted by the onset of symptoms [58]. This variability in joint ROM can influence the mechanical load on a determined part of the peripheral nervous system, depending on how the test is performed. This may offer an explanation for the differences observed between our findings and those from previous research [15,17].

Furthermore, moving joints through different ranges may expose specific neural segments to a greater external pressure [59] and a higher tension for a longer time period [15,17]. These mechanisms could help to justify the clinical relevance of neurodynamic sequencing and support its application in clinical practice.

### Limits of the Study

Several aspects must be considered when interpreting the results of this study.

First, peripheral nerves are thin, layered structures with anisotropic properties. For structures with these characteristics, the relationship between SWV and Young’s modulus can only be considered an estimate [60]. Also, in tissues that are relatively stiff and thin in comparison to the shear wavelength, the guided wave propagation can possibly bias SWV measurements [61].

In this study, all ULNT1 sequences did not modify the baseline nerve thickness, except for a minor difference in MN thickness observed between P0 and ULNT1-STD. Comparisons of neural structures in the same position showed that C6 was thicker than both C5 and the MN in the ULNT1-DIST and ULNT1-PROX sequences. No significant differences were detected between the other positions. Despite the slight differences mentioned above, nerve thickness was not correlated with SWV. Therefore, we believe that guided waves have not considerably contributed to bias in our results.

Furthermore, considering the anisotropic nature of nerves, the angle formed by the nerve relative to the ultrasound probe may have influenced elastographic measurements. In order to reduce this possibility, we placed the probe as perpendicular to the nerve as possible in all images. ULNT1 sequences did not alter the nerve angle at the same location, with the exception of a small difference (less than 2°) at C5 between the ULNT1-STD and ULNT1-PROX sequences. However, at the end of each sequence, C6 exhibited a greater angle than both C5 and MN, and C5 showed a greater angle than MN. According to previous research, structures with higher inclination angles relative to the probe may produce slightly lower SWE values than those oriented perpendicularly [62,63]. In our study, nerve angle was weakly negatively correlated with SWV; thus, when comparing different neural structures at the same position, there could be a small underestimation of SWV at the BP relative to the MN. On the other hand, nerve angle did not seem to significantly influence comparisons of the effect of different ULNT1 sequences at the same location.

Additionally, because the ROM was only measured for the final joint in each sequence, it is possible that other joints moved through different ranges. However, since we only included participants with no joint limitations and a similar nerve mechanosensitivity, any variations in mechanical forces on neural tissue due to different joint ranges are likely to be minimal.

Moreover, the starting position used in this study may not have fully unloaded the nervous system uniformly. Therefore, we cannot conclude whether the baseline differences in SWV between the BP and MN were due to spatial variations in nerve characteristics [36,37,64] or due to a different pre-tension of a determined neural segment. Future research is required to better understand how the different combinations of joint positions affect BP and MN stiffness.

Lastly, joint movements may have elongated the surrounding tissues and/or altered their position relative to the nerve, which could potentially have influenced the shear wave propagation during ultrasound elastography. The stretch of the myotendinous structures could have also caused lateral forces that further affected nerve length and local stiffness.

These effects are difficult to separate from the increase in neural tension produced by joint positioning and should be carefully considered when interpreting the results.

## 5. Conclusions

This study investigated the impact of three sequences of the ULNT1 on BP and MN stiffness using ultrasound SWE. The ULNT1-PROX resulted in the highest SWV at BP, while the ULNT1-STD and ULNT1-DIST produced significantly higher SWV distally at the MN. These findings suggest that BP and MN stiffness can vary with the order of joint movements during neurodynamic testing. However, since the effect of the surrounding tissues may have influenced nerve SWV, the results of the present study must be interpreted cautiously.

## Figures and Tables

**Table 1:** Intra-rater reliability of SWE measurements.

	ICC	SEM	MDC
Median nerve			
P0	0.933	0.13	0.37
ULNT1-STD	0.894	0.28	0.79
ULNT1-PROX	0.699	0.49	1.37
ULNT1-DIST	0.863	0.29	0.81
C5			
P0	0.973	0.12	0.35
ULNT1-STD	0.933	0.20	0.55
ULNT1-PROX	0.911	0.33	0.90
ULNT1-DIST	0.926	0.20	0.57
C6			
P0	0.962	0.16	0.45
ULNT1-STD	0.965	0.19	0.52
ULNT1-PROX	0.940	0.20	0.54
ULNT1-DIST	0.963	0.19	0.52

ICC = intraclass correlation coefficient. SEM = standard error of measurement. MDC = minimal detectable change.

**Table 2:** Ultrasound measurement data at each location and position.

		P0	ULNT1-STD	ULNT1-PROX	ULNT1-DIST
Median nerve	SWV (m/s)	2.76 ± 0.61	5.67 ± 0.91	4.13 ± 0.86	5.65 ± 0.98
	Thickness (mm)	2.35 ± 0.43	2.64 ± 0.44	2.60 ± 0.43	2.52 ± 0.40
	Angle (°)	5.51 ± 2.66	5.92 ± 1.88	5.84 ± 3.38	5.60 ± 2.02
C5	SWV (m/s)	3.28 ± 0.69	3.83 ± 0.83	4.88 ± 1.23	4.02 ± 0.86
	Thickness (mm)	2.36 ± 0.60	2.49 ± 0.68	2.57 ± 0.56	2.29 ± 0.44
	Angle (°)	13.72 ± 2.86	13.86 ± 3.09	11.94 ± 4.37	12.45 ± 4.65
C6	SWV (m/s)	3.77 ± 0.55	4.05 ± 0.82	4.87 ± 0.81	4.35 ± 0.98
	Thickness (mm)	2.70 ± 0.60	2.75 ± 0.44	2.94 ± 0.55	2.94 ± 0.62
	Angle (°)	14.71 ± 3.21	16.43 ± 5.24	15.13 ± 6.45	14.76 ± 4.88

**Table 3:** ULNT1 end-stages with ROM of the last joint moved.

ULNT1-STD	ULNT1-PROX	ULNT1-DIST
*N* = 31 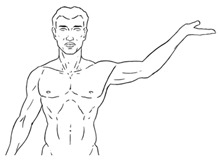 End-stage: elbow extension. ROM (°): 134.45 ± 13.75.	*N* = 31 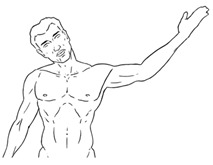 End-stage: elbow extension. ROM (°): 136.00 ± 19.18.	*N* = 30 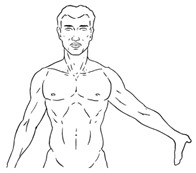 End-stage: shoulder abduction. ROM (°): 50.83 ± 14.38.
		*N* = 1 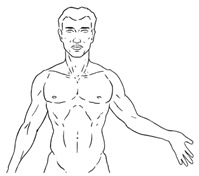 End-stage: elbow extension. ROM (°): 155.

ROM = range of motion.

## Data Availability

The datasets presented in this article are not readily available because the data are part of an ongoing doctoral thesis. Requests to access the datasets should be directed to the corresponding author.
